# Human Pol II promoter recognition based on primary sequences and free energy of dinucleotides

**DOI:** 10.1186/1471-2105-9-113

**Published:** 2008-02-24

**Authors:** Jian-Yi Yang, Yu Zhou, Zu-Guo Yu, Vo Anh, Li-Qian Zhou

**Affiliations:** 1 School of Mathematics and Computational Science, Xiangtan University, Hunan 411105, China; 2 School of Mathematical Sciences, Queensland University of Technology, GPO Box 2434, Brisbane, Q 4001, Australia

## Abstract

**Background:**

Promoter region plays an important role in determining where the transcription of a particular gene should be initiated. Computational prediction of eukaryotic Pol II promoter sequences is one of the most significant problems in sequence analysis. Existing promoter prediction methods are still far from being satisfactory.

**Results:**

We attempt to recognize the human Pol II promoter sequences from the non-promoter sequences which are made up of exon and intron sequences. Four methods are used: two kinds of multifractal analysis performed on the numeric sequences obtained from the dinucleotide free energy, Z curve analysis and global descriptor of the promoter/non-promoter primary sequences. A total of 141 parameters are extracted from these methods and categorized into seven groups (methods). They are used to generate certain spaces and then each promoter/non-promoter sequence is represented by a point in the corresponding space. All the 120 possible combinations of the seven methods are tested. Based on Fisher's linear discriminant algorithm, with a relatively smaller number of parameters (96 and 117), we get satisfactory discriminant accuracies. Particularly, in the case of 117 parameters, the accuracies for the training and test sets reach 90.43% and 89.79%, respectively. A comparison with five other existing methods indicates that our methods have a better performance. Using the global descriptor method (36 parameters), 17 of the 18 experimentally verified promoter sequences of human chromosome 22 are correctly identified.

**Conclusion:**

The high accuracies achieved suggest that the methods of this paper are useful for understanding the difficult problem of promoter prediction.

## Background

Promoter region plays an essential role in determining where the transcription of a particular gene should be initiated. Hence, promoter recognition – the computational task of finding the promoter regions on a DNA sequence, is an important problem [1]. The accumulation of a huge amount of genome sequence data in recent years makes the annotation process more and more complicated for higher eukaryotes [2]. The RNA polymerase II (Pol II) promoter is a key region that regulates differential transcription of protein coding genes. Computational analysis of Pol II promoters may contribute to improved gene identification and to prediction of the expression context of genes [3]. There is a need for prediction techniques that can rapidly and accurately evaluate sequences for the presence of promoter sequences [1].

Existing promoter prediction methods are still far from being satisfactory [3-5]. The performance of many current eukaryote promoter prediction methods has been unreliable with poor specificity or poor sensitivity [1]. Many methods predict promoter sequences based on the regulatory sequence elements (RSEs) in them. But the RSEs are short and not fully conserved in the promoter sequences, which results in a high probability of finding similar sequence elements elsewhere in genomes, outside the promoter regions. That is why most of the promoter prediction methods end up predicting a lot of false positions [6]. Fickett and Hatzigeorgiou [3] performed an evaluation of the different promoter prediction methods on genome DNA and suggested that it would be worth attempting nonlinear recognition methods, such as neural nets or quadratic discriminant analysis. Following this direction, Gangal and Sharma [7] applied time series descriptors and machine learning methods to human Pol II promoter prediction and got a higher accuracy compared with other methods; Kanhere and Bansal [6] presented a novel prokaryotic promoter prediction method based on DNA stability showing that the changing in the stability of DNA provides a much better clue than the usual sequence motifs.

In this paper, we attempt to recognize the human Pol II promoter sequences from the non-promoter sequences which contain exon and intron sequences. It should be noted that the aim of the present paper is similar to that of Ref. [7], but the non-promoter sequences in Ref. [7] are made up of coding sequences (CDSs) and intron sequences, while we use an existing database, the Exon/Intron database, to extract non-promoter sequences. We first convert the promoter/non-promoter sequences into numeric sequences according to the 10 unified free energy parameters [8], which have been used to measure the stability of DNA [6]. Then a measure representation is introduced for the numeric sequences. Multifractal analysis of the measure is next performed, which results in the first 5 parameters. Analogous multifractal analysis [9] is also used on the numeric sequences to achieve another 4 parameters. The Z curve method, which has been used in recent years with some successes [10,11], yields 96 parameters for the promoter/non-promoter primary sequences. The protein-chain descriptor method was first proposed by Dubchak *et al*. [12] to predict protein folding classes. Here we propose a global descriptor for the promoter/non-promoter sequences, which yields 36 parameters for a global description of the primary sequences. Overall, a total of 141 parameters are extracted from these four different methods and categorized into seven groups (methods). Fisher's linear discriminant algorithm shows that the global descriptor method is the most effective when used separately. Complete enumerations of all the possible combinations of these seven methods (120) are tested to find possibly better results with a relatively smaller number of parameters. Numerical results show that the methods with 96 and 117 parameters can produce satisfactory results. Compared with five other existing tools, the higher sensitivity, specificity, accuracy and correlation coefficient demonstrate that the methods proposed here are useful for understanding the human Pol II promoter prediction problem. 17 of the 18 experimentally verified promoter sequences of human chromosome 22 [13] are successfully identified by the global descriptor method (with only 36 parameters).

## Results

### Testing

We use two different data sets downloaded from two databases. The first set is the human Pol II promoter sequences from Release 90 of the Eukaryotic Promoter Database (EPD) [14]. The EPD is an annotated non-redundant collection of eukaryotic Pol II promoters, experimentally defined by a transcription start site (TSS) [15]. The EPD is a useful database when one wants to deal with the Pol II promoter prediction problem and it is broadly tested by different prediction tools [7,16-19]. A total of 1871 entries of human Pol II promoter sequences with window size of 499 bp upstream and 100 bp downstream of TSS, which is the same as that used in Ref. [16], are obtained from EPD. The sequences containing 'N' are manually filtered out, which results in a total of 1856 sequences. The second set is the non-promoter sequences of the human genome. For this data set, we consider using the Exon/Intron Database (EID), which incorporates information on the exon/intron structure of eukaryotic genes [20] ([21], hs35p1.EID.tar.gz). Firstly, the exon/intron sequences with 'n' and length less than 600 are filtered out. Then, we randomly select 1000 intron sequences from the file hs35p1.intrEID and 500 exon sequences from the file hs35p1.exEID. A fragment of length 600 is then selected randomly from each exon/intron sequence with length larger than 600. As the intron sequences are represented by lower-case letters in the file hs35p1.intrEID, we transform them into upper-case letters to be consistent with the promoter and exon sequences.

From the four different methods described in the Methods section, we get a total of 141 parameters. We will test their contributions in the promoter/non-promoter problem. Then we will try to combine some of them to see whether better results can be achieved.

For comparison of various methods, a benchmark should be set up. We use Fisher's linear discriminant algorithm [22-24] to calculate the discriminant accuracies. We divide all promoter and non-promoter sequences into two sets randomly. A set of 90% of promoter/non-promoter sequences is regarded as a training set, and the set of remaining 10% of promoter/non-promoter sequences as a test set.

Fisher's discriminant algorithm is used to find a classifier in the parameter space for a training set. The given training set *H *= {**x**_**1**_, **x**_**2**_, ⋯, **x**_**n**_} is partitioned into *n*_1 _≤ *n *training vectors in a subset *H*_1 _and *n*_2 _≤ *n *training vectors in a subset *H*_2_, where *n*_1 _+ *n*_2 _= *n *and each **x**_**i **_is a *κ*-dimensional vector, represented by one point in the *κ*-dimensional parameter space. Then *H *= *H*_1 _∪ *H*_2_. We need to find a parameter vector **w **= (*w*_1_, *w*_2_, ⋯, *w*_*κ*_)^*T *^for the *κ*-dimensional space such that ${\left\{y_{i}=wx_{i}\right\}}_{i=1}^{n}$ can be classified into two classes in the space of real numbers. If we denote

(1) $$\begin{array}{cc} m_{j}=\frac{1}{n_{j}}\sum_{x_{i}\in H_{j}}x_{i} & j=1,2, \end{array}$$

(2) $$\begin{array}{cc} S_{j}=\sum_{x_{i}\in H_{j}}(x_{i}-m_{j}){\left(x_{i}-m_{j}\right)}^{T}, & j=1,2, \end{array}$$

(3) **S**_*w *_= **S**_1 _+ **S**_2_,

then the parameter vector **w **is estimated as $S_{w}^{-1}(m_{1}-m_{2})$[23]. As a result, Fisher's discriminant rule becomes: "assign **x **to *H*_1 _if $Z(x)={\left(m_{1}-m_{2}\right)}^{T}S_{w}^{-1}[x-\frac{1}{2}(m_{1}+m_{2})]>0$ and to *H*_2 _otherwise" [22].

The discriminant accuracies for resubstitution analysis are defined as

(4) $$p_{c}=\frac{\text{The number of all correct promoter discriminations}}{\text{The number of promoter sequences in the training set}},$$

(5) $$p_{nc}=\frac{\text{The number of all correct non-promoter discriminations}}{\text{The number of non-promoter sequences in the training set}}.$$

For the test analysis, the discriminant accuracies *q*_*c *_and *q*_*nc *_are defined similarly by changing "training set" to "test set" in Eqs. (4) and (5), respectively.

We first divide the data into training and test sets randomly, then we use the above algorithm to calculate the discriminant accuracies for different methods. The results are listed in Table 1.

**Table 1:** The discriminant accuracies for various methods with Fisher's discriminant. The method marked "3+6+7" in the 8^th ^row means the combination of the methods listed in the 3^rd^, 6^th ^and 7^th ^rows. The meanings of the methods marked for the 9^th ^row is similar.

Order	*p*_*c*_(%)	*p*_*nc*_(%)	*q*_*c*_(%)	*q*_*nc*_(%)	Method	No. of parameters
1	73.05	85.63	74.73	83.33	*MFA*+*AMFA*	9
2	79.16	75.78	76.88	62.67	*ZC *Eq.(19)	9
3	78.86	88.00	79.03	85.33	*ZC *Eq.(21), *k *= 12	12
4	78.62	89.33	79.57	89.33	*ZC *Eq.(21), *k *= 23	12
5	80.30	90.74	80.65	90.00	*ZC *Eqs.(20, 22)	15
**6**	**85.75**	**88.30**	**86.02**	**91.33**	**GD**	**36**
7	81.92	91.48	81.72	89.33	*ZC *Eq.(23)	48
**8**	**86.11**	**93.48**	**86.02**	**90.67**	**3+6+7**	**96**
**9**	**86.89**	**93.11**	**86.02**	**92.67**	**1+3+4+6+7**	**117**
10	87.31	93.19	86.02	92.00	All methods	141

Firstly, seven groups of parameters are derived from the four methods: (i) 9 parameters from fractal methods (*MFA *and *AMFA*); (ii) 9 parameters from *ZC *representing the codon-position-dependent frequencies of mononucleotides; (iii) 12 parameters from *ZC *representing the frequencies of phase-specific dinucleotides (codon positions 1–2); (iv) 12 parameters from *ZC *representing the frequencies of phase-specific dinucleotides (codon positions 2–3); (v) 15 parameters for the phase-independent mononucleotides and dinucleotides from *ZC*; (vi) 36 parameters from *GD*; (vii) 48 parameters for the frequencies of phase-independent tri-nucleotides from *ZC*. From Table 1, it is seen that the results from the multifractal analyses seem to be better than that from *ZC *with an equal number of parameters, namely 9. We have successfully applied multifractal analyses in the clustering of large protein structures [9,25] and the distinction of coding and non-coding sequences in complete genomes [26], where the length of protein sequences and coding and non-coding sequences are larger than 300. It is well-known that the promoter sequences are highly diverse, which makes it notoriously difficult to generate patterns and rules for promoter prediction. It is expected that multifractal analyses can unfold some useful information on promoter sequences. The results from the frequencies of phase-specific dinucleotides at codon positions 2–3 in *ZC *indicate a better performance than that at codon positions 1–2. In addition, the accuracies from *ZC *with the frequencies of phase-independent mononucleotides and dinucleotides are improved but the number of parameters is increased to 15. The *GD *method shown in boldface in Table 1, denoted as **M1**, turns out to be especially useful as the accuracies are all larger that 85%. Compared with this, the results from the 48 parameters in *ZC *are not as good even though the number of parameters is increased.

Secondly, we want to test whether the results can be improved by increasing the number of parameters. It is not possible to test all the subsets of the 141 parameters but we can test all the combinations of the above seven methods (120 altogether). In our test, the accuracies do not simply increase as the number of parameters becomes larger, which indicates there might be some redundancy/correlation among the 141 parameters. For example, the accuracies with the 141 parameters are similar to those with only 117 parameters, suggesting the information from the mononucleotides and phase-independent dinucleotides in *ZC *is contained in the other methods. Therefore, all these parameters are not really needed. Nevertheless, in some circumstances the results do improve when the number of parameters is increased. Especially, among the 120 combinations, the results are relatively satisfactory in the cases of 96 and 117 parameters, which is shown in boldface in Table 1. We denote them by **M2 **and **M3**, respectively. In order to see whether multifractal analysis brings out useful information, we remove the 9 parameters of *MFA *and *AMFA *from **M3 **and test the results for such new combination. The *p*_*c*_, *p*_*nc*_, *q*_*c*_, and *q*_*nc *_calculated from this combination are: 86.05% 92.67%, 86.02% and 92.00% respectively. They are similar to those from **M3 **(86.89%, 93.11%, 86.02% and 92.67%), which demonstrates that multifractal analysis does not significantly improve the performance in **M3**.

In order to evaluate the correct prediction rate and reliability of a predictive method, the sensitivity (*S*_*n*_), specificity (*S*_*p*_), accuracy (*A*_*c*_) and correlation coefficient (*CC*) are also used [1]:

(6) *S*_*n *_= *T P/(T P + F N)*,

(7) *S*_*p *_= *T P/(T P + F P)*,

(8) *A*_*c *_= (*S*_*n*_*+ S*_*p*_)/2,

(9) $$CC=\frac{\left(TP\times TN)-(FP\times FN\right)}{\sqrt{\left(TP+FP)\times (TN+FN)\times (TP+FN)\times (TN+FP\right)}},$$

where *TP *denotes the number of correctly recognized promoter sequences, *FN *the number of promoter sequences recognized as non-promoter sequences, *FP *the number of non-promoter sequences recognized as promoter sequences, *TN *the number of correctly recognized non-promoter sequences.

From Fisher's discriminant algorithm, we calculate the four quantities defined above. The results related to Table 1 by the "order" mark are listed in Table 2.

**Table 2:** The accuracies of the prediction for promoter sequences by Fisher's discriminant algorithm. The *S*_*n*_, *S*_*p*_, *A*_*c *_and *CC *are the results for the training set and *S'*_*n*_, *S'*_*p*_, *A'*_*c*_ and *CC' *are the results for the test set. The rows are related to those in Table 1 according to the mark order.

Order	*S*_*n*_(%)	*S*_*p*_(%)	*A*_*c*_(%)	*CC*	${S^{\prime }}_{n}$(%)	${S^{\prime }}_{p}$(%)	${A^{\prime }}_{c}$(%)	*CC'*
1	73.05	86.28	79.67	0.58	74.73	84.76	79.74	0.58
2	79.16	80.17	79.67	0.55	76.88	71.86	74.37	0.40
3	78.86	89.05	83.95	0.66	79.03	86.98	83.01	0.64
4	78.62	90.12	84.37	0.68	79.57	90.24	84.91	0.69
5	80.30	91.47	85.89	0.71	80.65	90.91	85.78	0.70
**6**	**85.75**	**90.06**	**87.91**	**0.74**	**86.02**	**92.49**	**89.25**	**0.77**
7	81.92	92.25	87.08	0.73	81.72	90.48	86.10	0.71
**8**	**86.11**	**94.23**	**90.17**	**0.79**	**86.02**	**91.95**	**88.99**	**0.76**
**9**	**86.89**	**93.98**	**90.43**	**0.80**	**86.02**	**93.57**	**89.79**	**0.78**
10	87.31	94.06	90.68	0.80	86.02	93.02	89.52	0.78

Overall, from Tables 1 and 2, when the methods are used independently, we can see that **M1 **is the best one. The combined methods **M2 **and **M3 **improve the results. However, the number of parameters is too high in **M3**. Taking this aspect into account, a preferred method would be **M1 **or **M2**.

## Discussion

It is natural to ask whether the method of this paper has a better performance than the existing methods. As was done in Ref. [7], we can compare the present method with five kinds of promoter prediction tools, which are available on-line, namely Neural Network Promoter Prediction (NNPP version 2.2) [27], Soft Berry (TSSW) [28], Dragon Promoter Finder version 1.5 (DFP) [17,29], Promoter 2.0 [18,30] and Promoter Scan version 1.7 [19,31]. To be within a reasonable workload, we only compare with 10% of the promoter and non-promoter sequences used in Section 4 (186 promoter and 150 non-promoter sequences). The results are listed in Table 3. They clearly indicate that our method has a better performance than the other tools.

**Table 3:** The promoter prediction accuracies for the test data set made up of 186 promoter sequences and 150 non-promoter sequences using five kinds of tools and our methods.

Tool	*S*_*n*_(%)	*S*_*p*_(%)	*A*_*c*_(%)	*CC*
NNPP(threshold 0.8)	69.89	60.75	65.32	0.14
Soft Berry(TSSW)	67.74	81.29	74.52	0.48
Promoter Scan version 1.7	67.20	88.65	77.93	0.57
Dragon Promoter Finder version 1.5	30.65	65.52	48.08	0.12
Promoter 2.0 Prediction Server	52.15	91.51	71.83	0.49
Our method (**M3)**	86.02	93.57	89.79	0.78

However, using 90% of promoter sequences as a training set and only 10% of the promoter sequences as a test set may not provide a fair comparison against these methods. A more realistic performance would be to use 50% of the promoter sequences as a training set and the other 50% as a test set. Therefore, we use such ratio of training and test sets in Fisher's algorithm to see whether the results from our method are still satisfactory. We list the results of **M1**, **M2 **and **M3 **in Table 4. It shows that, with a smaller size of training set, the accuracy *A*_*c *_for the test set is surprisingly better than before, suggesting that our method is robust.

**Table 4:** The accuracies for M1, M2 and M3 with 50% sequences as training and the remaining 50% as test set in Fisher's discriminant algorithm.

Order	*S*_*n*_(%)	*S*_*p*_(%)	*A*_*c*_(%)	*CC*	${S^{\prime }}_{n}$(%)	${S^{\prime }}_{p}$(%)	${A^{\prime }}_{c}$(%)	*CC'*
**M1**	81.67	89.53	87.60	0.73	91.49	85.50	88.49	0.73
**M2**	87.28	93.32	90.30	0.79	90.41	89.07	89.74	0.77
**M3**	88.25	93.17	90.71	0.80	90.52	89.74	90.13	0.78

Based on support vector machine (SVM), Gangal and Sharma [7] used time series descriptors to identify promoter sequences from non-promoter sequences. They reported an accuracy of more than 85%. It will be interesting to see whether their method also works well in our test data set. But their tool Prometheus is not currently available. So it is not feasible to compare the two methods using the same data set. Nevertheless, by using 80% of data to train and the other 20% to test our method, which is the ratio used by Gangal and Sharma [7], we are able to produce a rough comparison with the results Gangal and Sharma reported (*S*_*n *_= 86% and *S*_*p *_= 88%). It is listed in Table 5, which shows that our results (*S*_*n *_= 87.10% and *S*_*p *_= 91.78%) are relatively better.

**Table 5:** The accuracies for M1, M2 and M3 with 80% sequences as training and the remaining 20% as test set in Fisher's discriminant algorithm.

Order	*S*_*n*_(%)	*S*_*p*_(%)	*A*_*c*_(%)	*CC*	${S^{\prime }}_{n}$(%)	${S^{\prime }}_{p}$(%)	${A^{\prime }}_{c}$(%)	*CC'*
**M1**	85.78	89.65	87.71	0.73	87.10	88.28	87.69	0.73
**M2**	86.39	93.71	90.05	0.79	87.90	91.09	89.49	0.77
**M3**	86.86	93.88	90.37	0.79	87.10	91.78	89.44	0.77

Finally, it is important to test our method with real human DNA sequences. For example, a sliding window technique with window size of 600 bp and step size of 10 bp can be used to recognize promoter sequences in the human DNA sequences, similar to the technique adopted by Gao and Zhang [32] to recognize exons. However, because promoter sequences are not clearly marked in the human DNA sequences, we can't use this approach to test our method. Nevertheless, similar to that performed in Ref. [7,33], we use the human chromosome 22, in which 20 promoters are experimentally verified [13]. One can refer to Table 1 in Ref. [13] to get the sequences with the accession numbers. However, as AB016655 and D86746 are not clearly annotated, we do not use them in the test. We use 50% of the promoter (from EPD) and non-promoter (from EID) sequences to train **M1**. The coefficients in Fisher's algorithm **w **= (*w*_1_, *w*_2_, ⋯, *w*_36_) are determined based on the training set. The choice of a promoter/non-promoter sequence is determined by the criterion **Z**(**x**) > **0**/**Z**(**x**) <**0**. Except for AF047576, the other 17 promoter sequences are correctly identified. This suggests that the global descriptor *GD *(**M1**), with a smaller number of parameters (36), is a practical method.

## Conclusion

Promoter prediction is a difficult but important problem in gene finding, and it is critical for elucidating the regulation of gene expression [34]. We use two kinds of multifractal analysis on the free energy sequences of promoter/non-promoter, Z curve analysis, and the global descriptor for the primary sequences of promoter/non-promoter. A total of 141 parameters are extracted from these four methods. These parameters are used in both independent and combined ways to distinguish promoter sequences from non-promoter sequences.

Fisher's linear discriminant algorithm provides a quantitative assessment of the recognition methods. If we use these methods independently, the global descriptor of the promoter/non-promoter sequences is the best method based Fisher's algorithm. Combinations of various methods show that the accuracies can be improved in some cases but the improvements are not simply due to the increase of parameter numbers. With all 141 parameters together, the results are satisfactory. However, the number of parameters is too high in this condition. The number is reduced as there is some redundancy/correlation among these parameters. In the case of 117 parameters, similar results are achieved, with the discriminant accuracies *p*_*c*_, *p*_*nc*_, *q*_*c*_, and *q*_*nc *_reaching 86.89% 93.11%, 86.02% and 92.67%, respectively. The related sensitivity *S*_*n*_, specificity *S*_*p*_, accuracy *A*_*c *_and correlation coefficient *CC *for the test set reach 86.02%, 93.57%, 89.79% and 0.78, respectively. A smaller number of parameters (96) also produces relatively satisfactory results. The global descriptor method with only 36 parameters successfully identifies 17 of the 18 experimentally verified promoters in human chromosome 22 [13]. Recognition of promoter sequences with such satisfactory accuracy indicates that the methods is promising for human Pol II promoter prediction.

The main aim of this work is to develop efficient algorithms that can discriminate between promoters and non-promoters in a given sequence. Another challenge being addressed is the localization of promoters rather than a simple classification considered in current methods [7]. Multifractal analysis, which is especially useful in many other fields [25,26,35,36], seems to reflect some information for promoter recognition (see first line in Table 1). But if we use method **M3**, multifractal analysis does not significantly improve the performance. The methods considered in this paper seem promising in enhancing the performance of biomolecular sequence analysis and promoter prediction in particular. It is a challenge to predict promoter sequences directly from the real human genome. However, it would be helpful to use first the ENCODE pilot project data set, which spans about 1% of the human genome sequence [37]. Our following work aims to contribute towards this challenging problem.

## Methods

### Conversion of the original data

**Figure 1:**
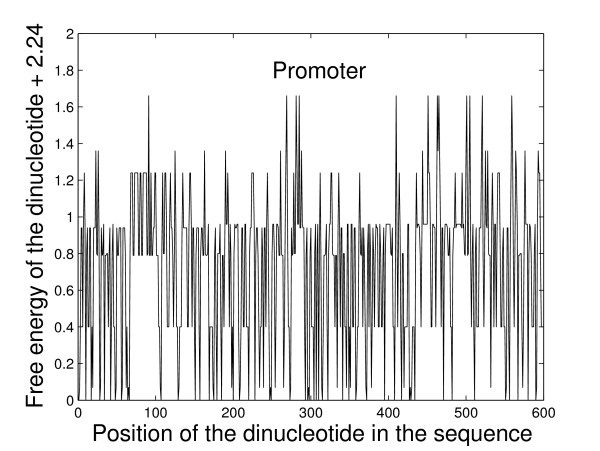
**The free energy sequence of one promoter sequence.** See text for a detailed description about how to get such numeric sequence.

Some studies suggested that various properties, such as stability, bendability and curvature, of the region immediately upstream of the TSS differ from that of downstream region [6,38,39]. The upstream region is less stable, more rigid and more curved than the downstream region. Kan-here and Bansal [6] predicted the prokaryotic promoter based on such difference in DNA stability. We convert the original sequences into new numeric sequences according to the free energy of dinucleotides. A sliding window with size of 2nt is used and moved one base pair forward each time. The numeric sequences can be smoothed with a larger window size. For more details on the smoothing method, one can refer to Ref. [40]. The free energy values corresponding to the 10 unique dinucleotides are taken from the unified parameters proposed in Ref. [8]. They are: AA/TT = -1.00 *kcal/mol*, AT/TA = -0.88 *kcal/mol*, TA/AT = -0.58 *kcal/mol*, CA/GT = -1.45 *kcal/mol*, CT/GA = -1.44 *kcal/mol*, GT/CA = -1.28 *kcal/mol*, GA/CT = -1.30 *kcal/mol*, CG/GC = -2.17 *kcal/mol*, GC/CG = -2.24 *kcal/mol*, GG/CC = -1.84 *kcal/mol*. The ten values are added by 2.24 *kcal/mol *(the negative of the smallest free energy) so that all the values are larger than or equal to zero in order to construct a measure from the time series for the multifractal method in the following analysis. For example, the free energy sequence for one of the promoter sequences with a sliding window of size 2nt is given in Figure 1.

### Multifractal analysis (*MFA*)

Let *T*_*t*_, *t *= 1, 2, ⋯, *N*, be the numeric sequence of a promoter/non-promoter with length *N *. First, we define

(10) $$\begin{array}{cc} F_{t}=\frac{T_{t}}{\sum_{j=1}^{N}T_{j}}, & \left(t=1,2,\cdots ,N\right) \end{array}$$

to be the frequency of *T*_*t*_. It follows that $\sum_{t=1}^{N}F_{t}=1$. We define a measure *μ *on the interval [0, 1) by

(11) *μ*(*dx*) = *Y*(*x*) *dx*,

where

(12) $$\begin{array}{cc} Y(x)=N\times F_{t}=\frac{T_{t}}{\frac{1}{N}\sum_{j=1}^{N}T_{j}}, & x\in [\frac{t-1}{N},\frac{t}{N}). \end{array}$$

We denote the interval $\left[\frac{t-1}{N},\frac{t}{N}\right)$ by *I*_*t*_. It is easy to see that *μ*([0, 1)) = 1 and *μ*(*I*_*t*_) = *F*_*t*_. We call *μ*(*x*) the *measure representation *[26,41] for the numeric sequence of a promoter/non-promoter.

The most common algorithms of multifractal analysis are the so called *fixed-size box-counting algorithms *[42]. In the one-dimensional case, for a given measure *μ *with support *E *⊂ ℝ, we consider the *partition sum*

(13) $$\begin{array}{cc} Z_{\varepsilon }(q)=\sum_{\mu (B)\neq 0}{\left[\mu (B)\right]}^{q}, & q\in \mathbb{R}, \end{array}$$

where the sum runs over all different nonempty boxes *B *of a given side *ε *in a grid covering of the support *E*, that is,

(14) *B *= [*kε*, (*k *+ 1)*ε*).

The *mass exponent τ *(*q*) is defined [43,44] as

(15) $$\tau (q)=\underset{\varepsilon \to 0}{\lim}\frac{\ln Z_{\varepsilon }(q)}{\ln\varepsilon }$$

and the generalized *fractal dimensions *[43,44] of the measure are defined as

(16) $$\begin{array}{ccc} D(q)=\frac{\tau (q)}{q-1}, & for & q\neq 1, \end{array}$$

and

(17) $$\begin{array}{ccc} D(q)=\underset{\varepsilon \to 0}{\lim}\frac{Z_{1,\varepsilon }}{\ln\varepsilon }, & for & q=1, \end{array}$$

where $Z_{1,\varepsilon }=\sum_{\mu (B)\neq 0}\mu (B)\ln\mu (B)$. The generalized fractal dimensions are numerically estimated through a linear regression of ln *Z*_*ε *_(*q*)/(*q *- 1) against ln *ε *for *q *≠ 1, and similarly through a linear regression of *Z*_1, *ε *_against ln *ε *for *q *= 1 [25,42,45]. *D*(1) is called the *information dimension *and *D*(2) the *correlation dimension *[43,44].

The concept of *phase transitions *in multifractal spectra was introduced in the study of logistic maps, Julia sets, and other simple systems. Evidence of a phase transition was found in the multifractal spectrum of diffusion-limited aggregation [46]. By following the thermodynamic formulation of multifractal measures, Canessa [47] derived an expression for the analogous specific heat as

(18) $$C_{q}\equiv -\frac{\partial^{2}\tau (q)}{\partial q^{2}}\approx 2\tau (q)-\tau (q+1)-\tau (q-1).$$

He showed that the form of *C*_*q *_resembles a classical phase transition at a critical point for financial time series.

The singularities of a measure are characterized by the *Lipschitz-Hölder exponent α*(*q*) [44], which is related to *τ *(*q*) by

(19) $$\alpha (q)=\frac{d}{dq}\tau (q).$$

Substitution of Eq. (15) into Eq. (19) yields

(20) $$\alpha (q)=\underset{\varepsilon \to 0}{\lim}\frac{\sum_{\mu (B)\neq 0}{\left[\mu (B)\right]}^{q}\ln\mu (B)}{Z_{\varepsilon }(q)\ln\varepsilon }.$$

Again, the exponent *α(q*) can be estimated through a linear regression of $\left\{\sum_{\mu (B)\neq 0}{\left[\mu (B)\right]}^{q}\ln\mu (B)\}/Z_{\varepsilon }(q\right)$ against ln *ε*. The multifractal spectrum *f *(*α*) versus *α *can be calculated according to a relationship known as *Legendre transformation *[44]:

(21) $$f(\alpha )=\underset{q}{\min}\{q\alpha (q)-\tau (q)\}.$$

**Figure 2:**
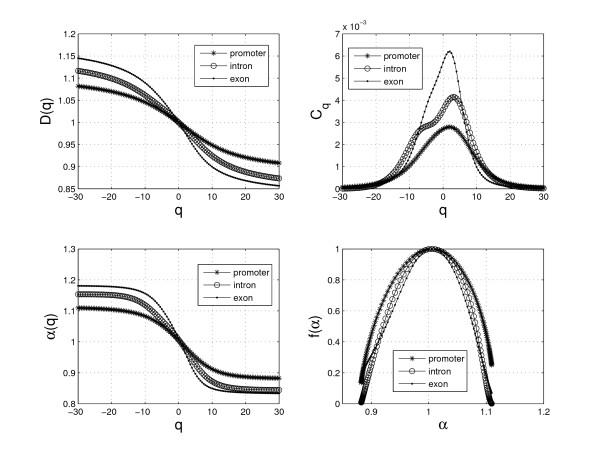
**The four kinds of fractal curves for the promoter, exon and intron sequences.** The figures show that there are some differences between the promoter and non-promoter (exon/intron) sequences, which suggests that it's possible to extract some values from them to distinguish the promoter sequences from the non-promoter sequences.

We first construct a measure for the numeric sequences according to Eq. (11), then analyze the measure with the above multifractal method. The *D*(*q*), *C*_*q*_, *α*(*q*) and *f *(*α*) curves for one of the promoter, exon and intron sequences are shown in Figure 2. We select 5 parameters from *MFA *to distinguish between promoter and non-promoter sequences: *D*(2), *C*_1_, *C*_*max *_(the maximum value of *C*_*q*_), Δ*α *= *α*_*max *_- *α*_*min *_and Δ*f *= *f *(*α*_*max*_) - *f *(*α*_*min*_).

### Analogous multifractal analysis (*AMFA*)

Analogous multifractal analysis is similar to *multiaffinity analysis *which is a useful method in many fields. It was recently proposed in [9]. We denote a time series as *X*(*t*), *t *= 1, 2, ⋯, *N*. First, the time series is integrated as

(22) $$\begin{array}{cc} {y^{\prime }}_{q}(k)=\sum_{t=1}^{k}{\left(X(t)-X_{ave}\right)}^{q}, & \left(q\in {\mathbb{Z}}_{+},k=1,2,\cdots ,N\right) \end{array}$$

(23) $$\begin{array}{cc} y_{q}(k)=\sum_{t=1}^{k}|X(t)-X_{ave}{|}^{q}, & \left(q\neq 0,k=1,2,\cdots ,N\right) \end{array}$$

where *X*_*ave *_is the average over the whole time period and *k *∈ [1, *N*]. Then two quantities *M*_*q *_(*L*) and ${M^{\prime }}_{q}(L)$ are defined as

(24) $$\begin{array}{cc} {M^{\prime }}_{q}(L)={\left[{\langle |y^{\prime }(j)-y^{\prime }(j+L)|\rangle }_{j}\right]}^{\frac{1}{q}}, & \left(q\in {\mathbb{Z}}_{+}\right) \end{array}$$

(25) $$\begin{array}{cc} M_{q}(L)={\left[{\langle |y(j)-y(j+L)|\rangle }_{j}\right]}^{\frac{1}{q}}, & \left(q\neq 0\right) \end{array}$$

where 〈〉_*j *_denotes the average over *j, j *= 1, 2, ⋯, *N *– *L*; *L *typically varies from 1 to *N*_1 _in which the linear fit is good. From the ln *L *vs ln *M*_*q *_(*L*) and ln *L *vs ln ${M^{\prime }}_{q}(L)$ planes, one can determine the relations:

(26) $$\begin{array}{ccc} {M^{\prime }}_{q}(L)\propto L^{h^{\prime }(q)} & for & q\in {\mathbb{Z}}_{+}, \end{array}$$

(27) $$\begin{array}{ccc} M_{q}(L)\propto L^{h(q)} & for & q\neq 0. \end{array}$$

Linear regressions of ln ${M^{\prime }}_{q}(L)$ and ln *M*_*q *_(*L*) against ln *L *will yield the exponents *h' *(*q*) and *h*(*q*) respectively.

The exponent *h*(*q*) has a nonlinear dependence on *q*. When *q *= 1, the methods are just those reported in Refs. [48,49] and these methods are used to study the length sequences from the complete genomes by Yu *et al*. [49]. *M'*(*L*) may be assessed to determine long-range correlation [50]. From Ref. [49], the linear fit to get the exponent *h*(1) is better than that to get the exponent *h'*(1). Our numerical results show that the exponents *h*(*q*) are more robust than the exponents *h'*(*q*), so we suggest to use the exponents *h*(*q*). We have used *h*(*q*) in clustering the structure of large proteins and it turns out to be a useful method [9].

**Figure 3:**
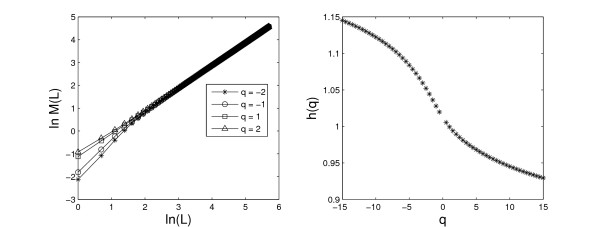
The relationship between ln *M*(*L*) and ln(*L*) using the free energy sequence of one promoter (Left); the h(q) spectra for the one promoter calculated by AMFA (Right).

Figure 3 gives an example in applying the AMFA to the free energy sequence of a promoter sequence. It shows a good linear relationship between ln *M*(*L*) and ln(*L*). For different values of *q*, we get the exponents *h*(*q*) from linear regressions of ln *M*(*L*) against ln (*L*) according to Eq. (27). The exponent spectrum *h*(*q*) of the promoter sequence is shown in the right panel of Figure 3. We extract four parameters from AMFA: *h*(-2), *h*(-1), *h*(1) and *h*(2).

### Z curve (*ZC*)

The concept of the Z curve representation of a DNA sequence was first proposed by Zhang and Zhang [51], and was used to distinguish coding and noncoding DNA sequences [52,53]. A new system based on *ZC*, Z CURVE 1.0, for finding protein-coding genes in bacterial and archaeal genomes has been proposed [10]. Recently, another new self-training system based on the *ZC *method, ZCURVE_V [11], for recognizing protein-coding genes in viral and phage genomes was reported.

In this paper, we apply the *ZC *method in distinguishing promoter and non-promoter sequences. For convenience, we give a brief description of the methods in Refs. [10] and [11]. The frequencies of bases A, C, G and T occurring in a promoter/non-promoter sequence with bases at positions 1, 4, 7, ⋯; 2, 5, 8, ⋯; 3, 6, 9, ⋯, are denoted by *a*_1_, *c*_1_, *g*_1_, *t*_1_; *a*_2_, *c*_2_, *g*_2_, *t*_2_; *a*_3_, *c*_3_, *g*_3_, *t*_3_, respectively. They are in fact the frequencies of bases at the first, second and third codon positions, which can be called *codon-position-dependent *frequencies of mononucleotides. Based on the *ZC *[54], *a*_*i*_, *c*_*i*_, *g*_*i*_, *t*_*i *_for each *i *can be used to construct three coordinates, denoted by *x*_*i*_, *y*_*i *_and *z*_*i *_according to the Z transform [54]:

(28) $$\{\begin{array}{l} x_{i}=(a_{i}+g_{i})-(c_{i}+t_{i}),\\ y_{i}=(a_{i}+c_{i})-(g_{i}+t_{i}),\\ z_{i}=(a_{i}+t_{i})-(g_{i}+c_{i}), \end{array}$$

where *x*_*i*_, *y*_*i*_, *z*_*i *_∈ [-1, 1], *i *= 1, 2, 3.

We can use the above 9 parameters in the promoter/non-promoter problem. We can also consider the *codon-position-independent *frequencies of single bases, which results in the following three coordinates:

(29) $$\{\begin{array}{l} x=(a+g)-(c+t),\\ y=(a+c)-(g+t),\\ z=(a+t)-(g+c), \end{array}$$

where *x, y, z *∈ [-1, 1], *a, c, g *and *t *are the frequencies for the bases A, C, G and T in a promoter/non-promoter sequence, respectively.

In addition to the frequencies of codon-position-dependent mononucleotide, we also consider the frequencies of *phase-specific *dinucleotides. We denote the frequencies of the 16 dinucleotides AA, AC, ⋯, and TT occurring at the codon positions 1–2 and 2–3 of a promoter or non-promoter sequence by *p*_12_(*AA*), *p*_12_(*AC*), ⋯, *p*_12_(*T T*); *p*_23_(*AA*), *p*_23_(*AC*), ⋯, and *p*_23_(*T T*), respectively. Using the Z transform [54], the following 24 coordinates can be defined:

(30) $$\{\begin{array}{l} x_{k}^{X}=(p_{k}(XA)+p_{k}(XG))-(p_{k}(XC)+p_{k}(XT)),\\ y_{k}^{X}=(p_{k}(XA)+p_{k}(XC))-(p_{k}(XG)+p_{k}(XT)),\\ z_{k}^{X}=(p_{k}(XA)+p_{k}(XT))-(p_{k}(XG)+p_{k}(XC)), \end{array}$$

where $x_{k}^{X},y_{k}^{X},z_{k}^{X}\in [-1,1]$*p*_*k *_(*XY*) = *n*_*k*_(*XY*)/[*n*_*k *_(*XA*) + *n*_*k *_(*XC*) + *n*_*k*_(*XG*) + *n*_*k *_(*XT*)], *n*_*k*_(*XY*) are the occurring times of dinucleotides XY, X, Y = A, C, G, T, *k *= 12, 23.

We can also consider the frequencies of phase-specific dinucleotides and the frequencies of *phase-independent *dinucleotides. For this purpose, a sliding window with size 2nt is used and moved forward one base each time to count the number of times of the occurring dinucleotides. With this method, 12 new coordinates can be defined:

(31) $$\{\begin{array}{l} x^{X}=(p(XA)+p(XG))-(p(XC)+p(XT)),\\ y^{X}=(p(XA)+p(XC))-(p(XG)+p(XT)),\\ z^{X}=(p(XA)+p(XT))-(p(XG)+p(XC)), \end{array}$$

where *x*^*X*^, *y*^*X*^, *z*^*X *^∈ [-1, 1], *p*(*XY*) = *n*(*XY*)/[*n*(*XA*) + *n*(*XC*) + *n*(*XG*) + *n*(*XT*)], *n*(*XY*) are the occurring times of dinucleotides XY, X, Y = A, C, G, T.

Gao and Zhang [32] compared various algorithms for recognizing short coding sequences of human genes and they defined 48 quantities, which were the frequencies of *phase-dependent *tri-nucleotides. In Ref. [32], Gao and Zhang used a sliding window with size 3nt and the window was moved forward three bases each time to count the frequencies for the 64 tri-nucleotides. Now we move forward the sliding window with size 3nt one base each time. The definition for the 48 coordinates is

(32) $$\{\begin{array}{l} x^{XY}=(p(XYA)+p(XYG))-(p(XYC)+p(XYT)),\\ y^{XY}=(p(XYA)+p(XYC))-(p(XYG)+p(XYT)),\\ z^{XY}=(p(XYA)+p(XYT))-(p(XYG)+p(XYC)), \end{array}$$

where *x*^*XY*^, *y*^*XY*^, *z*^*XY *^∈ [-1, 1], *p*(*XYZ*) = *n*(*XYZ*)/[*n*(*XYA*) + *n*(*XYC*) + *n*(*XYG*) + *n*(*XYT*)], *n*(*XY Z*) are the occurring times of trinucleotides XYZ, X, Y, Z = A, C, G, T. The difference between Ref. [32] and here is in the calculation of *n*(*XYZ*); the present method can be regarded as a *phase-independent *method.

### Global descriptor of promoter/nonpromoter sequence (*GD*)

Dubchak *et al*. [12] proposed a method for predicting protein folding classes based on a global protein chain description. The protein-chain descriptor includes overall composition, transition, and distribution of amino acid attributes. Similar methods have also been used in Refs. [55-58]. In this paper, we propose the global descriptor of promoter/non-promoter sequences.

The global description contains three parts: composition (*Comp*), transition (*Tran*) and distribution (*Dist*). In order to explain the method, we suppose that a sequence consists of only two kinds of letters (A and B). The composition is used to measure the frequency of occurrence of each kind of letters in the sequences. For example, for the sequence: BABBABABBABBAABABABBAAAB-BABABA, there are 14 As and 16 Bs, hence the frequencies for A and B are 100.00 × 14/(14+16) = 46.67, 100.00 × 16/(14+16) = 53.33, respectively. These two numbers represent the first part of the global description, *Comp*. The second part, *Tran*, characterizes the percent frequency with which A is followed by B or B is followed by A. For example, for the above sequence, there are 21 transitions of this type, that is, (21/29) × 100.00 = 72.14. The third part of the global description, *Dist*, measures the chain length within which the first, 25%, 50%, 75% and 100% of certain type of letters is located, respectively. For example, for the above sequence, the first, 25%, 50%, 75% and 100% of Bs are located within the first, 6th, 12th, 20th and 29th nucleotides, respectively. The *Dist *descriptor for Bs is thus: 1/30 × 100.00 = 3.33, 6/30 × 100.00 = 20.00, 12/30 × 100.00 = 40.00, 20/30 × 100.00 = 66.67 and 29/30 × 100.00 = 96.67. Likewise, the *Dist *descriptor for As is 6.67, 23.33, 53.33, 73.33 and 100.00. As a result, the global description for the above sequence is (*Comp*; *Tran*; *Dist*) = (46.67, 53.33; 72.14; 6.67, 23.33, 53.33, 73.33, 100.00, 3.33, 20.00, 40.00, 66.67, 96.67). A more detailed description of global description of sequences is given in Refs. [12,55-58].

The global description for the promoter/non-promoter sequences can be computed by a similar procedure. As the sequences consist of four types of nucleotides (A, C, G and T), there are 4 parameters for *Comp*, 6 parameters for *Tran *and 20 parameters for *Dist*. Overall, a total of 30 parameters are used to give a global description of a promoter/non-promoter sequence.

The Entropy Density Profile (EDP) model is a global statistical description for a DNA sequence, which employs Shannon's artificial linguistic description for a DNA sequence of finite length like an open reading frame (ORF) [59]. Zhu *et al*. [59] developed a new non-supervised gene prediction algorithm for bacterial and archaeal genomes based on EDP. Here we describe such method briefly. If *p*_*i*_(*i *= 1, 2, 3, 4) are the frequencies for the four types of nucleotides of a promoter/non-promoter sequence, then an EDP vector *S *= {*s*_*i*_} inferred from {*p*_*i*_} is used to represent the sequence with an emphasis on the information content, where *i *is the index of the four kinds of nucleotides. The EDP *s*_*i *_is defined as [59]

(33) $$\begin{array}{cc} s_{i}=-\frac{1}{H}p_{i}\log p_{i}, & i=1,2,3,4, \end{array}$$

where $H=-\sum_{i=1}^{4}p_{i}\log p_{i}$ is the Shannon entropy.

It was shown that $P=p_{1}^{2}+p_{2}^{2}+p_{3}^{2}+p_{4}^{2}$ is a useful statistical quantity for analysis of DNA sequences [54,60], which was called a nucleotide composition constraint of genomes [61]. As a result, we obtain 6 parameters *s*_1_, *s*_2_, *s*_3_, *s*_4_, *H *and *P *from EDP.

Overall, combining the above two description systems, we get 36 parameters for the global descriptor of a promoter/non-promoter sequence.

## Authors' contributions

JYY conceived of the study, downloaded the data, analyzed the results, has been involved in programming, drafting and revising the manuscript. YZ and LQZ have been involved in the programming and discussion on the results. ZGY coordinated the study and participated in its design, analyzed the results, has been involved in drafting and revising the manuscript. VA participated in the design of the study and the results discussion, has been involved in drafting and revising the manuscript. All authors read and approved the final manuscript.
